# Genome-wide identification and expression analysis of the MYB transcription factor family in *Desmodium styracifolium*

**DOI:** 10.3389/fpls.2026.1938247

**Published:** 2026-09-03

**Authors:** Jie Zhou, Jiaxin Wu, Mei Liu, Xiasheng Zheng

**Affiliations:** School of Pharmaceutical Science, Guangzhou University of Chinese Medicine, Guangzhou, Guangdong, China

**Keywords:** *Desmodium styracifolium*, evolution, MYB transcription factor, phylogeny, structure

## Abstract

**Introduction:**

The aerial parts of *Desmodium styracifolium* are widely used in traditional Chinese medicine to treat urinary calculi, with flavonoids as the primary active components. MYB transcription factors are key regulators of flavonoid biosynthesis in plants; however, the MYB family of *D. styracifolium* has not yet been systematically investigated.

**Methods:**

Using previously obtained whole-genome and transcriptome data, *DsMYB* genes were identified through combined hmmsearch and blastp screening. Phylogenetic relationships were reconstructed together with 131 *Arabidopsis thaliana* MYB proteins. Chromosomal localization, collinearity, conserved motifs, gene structure, and promoter cis-acting elements were analyzed. Expression patterns across tissues and under high-light treatment were profiled, and eight representative genes were verified by quantitative real-time PCR (qRT-PCR).

**Results:**

A total of 161 *DsMYB* genes were identified and categorized into R2R3-MYB (134), 1R-MYB (21), 3R-MYB (5), and 5R-MYB (1) subfamilies; 132 genes were unevenly distributed across nine chromosomes, with the remaining 29 located on unplaced scaffolds. Collinearity analysis indicated that segmental duplication was the primary driver of R2R3-MYB expansion. Phylogenetic analysis resolved 25 subgroups, among which the largest subgroup C13 (30 members) was specific to *D. styracifolium*, and subgroups C20 and C17 corresponded to the Arabidopsis anthocyanin and flavonol regulatory clades, respectively. Most DsMYB proteins shared core conserved motifs 2~5, and their promoters were enriched in light-, abscisic acid-, and methyl jasmonate-responsive elements. Eight qRT-PCR-verified *DsMYB* genes exhibited tissue-specific expression and distinct induction patterns under high light.

**Discussion:**

This study provides the first systematic characterization of the MYB family in *D. styracifolium*. The enrichment of light- and hormone-responsive cis-elements, together with the light-responsive expression of candidate members, implicates DsMYBs in the transcriptional control of flavonoid biosynthesis, with the C17 and C20 subgroups emerging as priority candidates for flavonol and anthocyanin regulation.

## 1 Introduction

*Desmodium styracifolium* (Osbeck) H.Ohashi & K.Ohashi, a leguminous plant commonly known as Guangdong Jinqiancao, plays a pivotal role in traditional Chinese medicine (TCM). Its dried aerial parts are frequently prescribed for the clinical treatment of urinary calculi (Cheng et al., 2018; Liu et al., 2017; Xiong et al., 2015). Due to its proven curative effect, this plant serves as a primary raw material for several Chinese patent medicines, highlighting its significant medicinal and economic value.

Chemical analyses indicate that *D. styracifolium* contains a diverse array of compounds, including flavonoids, alkaloids, tannins, and polysaccharides (Bora et al., 2023); however, flavonoids have been identified as the primary active components responsible for its therapeutic effect against urinary calculi. Notably, total flavonoids purified from this herb were developed into a Class 1.2 novel drug, which has been marketed in China since 2022. Furthermore, schaftoside, the most abundant flavonoid in this species, has been designated by the Chinese Pharmacopoeia as the official quality evaluation marker (Guo et al., 2015).

In addition to schaftoside, *D. styracifolium* contains related flavone C-glycosides such as isoschaftoside, vitexin, and orientin. These compounds are formed through the condensation of carbon atoms with pyranose hexoses or furanose pentoses on apigenin aglycone (Opryshko et al., 2024). Several coding genes for key enzymes in the biosynthetic pathway of these flavone C-glycosides, including CHS, CHI, FNS (Xie et al., 2023), F2H, and CGT (Li et al., 2024). While these studies established a framework for understanding flavonoids biosynthesis, the regulatory mechanisms governing these pathways remain poorly understood in *D. styracifolium*.

The biosynthesis of flavonoids is regulated by a diverse array of transcription factors, with the MYB (Myeloblastosis) family playing a pivotal role (Albert et al., 2018; Tong et al., 2024). Ubiquitous in plants, MYB transcription factors are involved in a wide range of biological activities. Their defining characteristic is a highly conserved DNA-binding domain, typically composed of approximately 52 amino acid residues with one to four incomplete repeats (Li et al., 2019). This domain facilitates the specific recognition and binding to *cis*-acting elements within the promoter regions of target genes.

According to the number of repeat units, MYB factors are systematically classified into four major categories: 1R-MYB (containing R1/R2 or R3-type repeats), 2R-MYB (R2R3-MYB type), 3R-MYB (R1R2R3-MYB type), and 4R-MYB (Millard et al., 2019). 1R-MYB proteins primarily regulate plant growth and stress responses, whereas R2R3-MYB factors are the most prevalent type, participating in cell morphology, pattern formation, and metabolism (Feller et al., 2011). While 3R-MYB proteins are associated with cell cycle regulation (Haga et al., 2007), 4R-MYB proteins are scarce and their functional roles have yet to be fully elucidated (Dubos et al., 2010). Existing research recognize the critical role of MYB factors in the metabolic pathway of flavonoids. For instance, genes such as *MdMYB114, MdMYB1* in *Malus domestica* positively regulate anthocyanin synthesis (Hu et al., 2016; Jiang et al., 2021); while *AtMYB11, AtMYB12*, and *AtMYB111* in *Arabidopsis thaliana* modulate the flavonoid content by controlling early biosynthetic genes like CHS and CHI (Luo et al., 2008; Pandey et al., 2014, 2015; Stracke et al., 2007). Furthermore, evidence suggested that the heterologous overexpression of *BsMYB36* in *Arabidopsis thaliana* increased flavonoid content, while reduced the number of anthocyanins and proanthocyanidins, simultaneously. Meanwhile, homologous overexpression of *BsMYB51* promoted the accumulation of flavonoids, anthocyanins, and proanthocyanidins (Huang et al., 2025).

Despite these advances, the molecular regulatory mechanism governing flavonoid biosynthesis in *D. styracifolium* remain poorly understood. This knowledge gap is particularly evident regarding the transcription factor regulatory network; specifically, the MYB family in this species has not yet been systematically identified or functionally analyzed. Therefore, the objective of the present study was to systematically identify all MYB family members using whole genome data. We characterized these genes through comprehensive bioinformatic analysis and predicted their potential functions via *cis*-acting element and expression profile analyses. Additionally, we screened core candidate genes that involved in the regulation of flavonoid biosynthesis. These results establish a theoretical foundation for future investigations into the molecular regulatory mechanisms of the phytochemical optimization of this herb.

## 2 Materials and methods

### 2.1 Plant materials

Samples of *D. styracifolium* were collected from the “Shizhenshan” Medicinal Garden of Guangzhou University of Chinese Medicine. A voucher specimen was deposited in the Herbarium of the Division of Chinese Medicinal Resources at the same institution. To preserve sample integrity, all tissues were immediately frozen in liquid nitrogen and stored at - 80 °C to RNA extraction and metabolite analysis. To ensure the reliability and reproducibility, three independent biological replicates were processed for each sample.

### 2.2 Genome-wide identification of *DsMYB* genes

The whole-genome sequence and gene annotations of *D. styracifolium* were retrieved from our previous study using third-generation sequencing technologies. To systematically identify all the members of *DsMYB* gene family, a dual-method screening strategy was employed. Initially, a Hidden Markov Model (HMM) search was performed using the MYB conserved domain file (PF00249) retrieved from the Pfam database (http://pfam.xfam.org/). Sequences with an E-value ≤ 1e-10 were retained. Subsequently, MYB protein sequences from *Arabidopsis thaliana* (TAIR10) (https://www.arabidopsis.org) were used as reference queries for a Blastp search against the *D. styracifolium* proteome. This search was conducted using Diamond software with an E-value cutoff of 1e-5 and a minimum identity threshold of 50%. The results from both methods were integrated and deduplicated. For structural validation, all candidate genes were submitted to the NCBI Batch CDD-search (www.ncbi.nlm.nih.gov/Structure/bwrpsb/bwrpsb.cgi). Any sequences lacking a complete MYB domain were manually excluded to establish the final set of *DsMYB* family members. The essential properties of DsMYB proteins were predicted by ExPASy (https://web.expasy.org/protparam/). In addition, the subcellular localization of *DsMYB* genes was predicted by the WolF PSORT (https://wolfpsort.hgc.jp/).

### 2.3 Sequence alignment and phylogenetic analysis

Multiple sequence alignment of the DsMYB amino acid sequences was performed using MAFFT. Based on these alignments, a phylogenetic tree was constructed via FastTree. Subsequently, the inferred phylogeny was visualized and refined using the EvolView (https://evolgenius.info//evolview-v2/) online platform.

### 2.4 Chromosomal localization and collinearity analysis

Chromosomal distribution of *DsMYB* genes was analyzed and visualized using TBtools, based on the *D. styracifolium* genome annotation. To characterize gene homology, the MCScan was employed to identify homologous MYB gene pairs between *D. styracifolium* and *Arabidopsis thaliana*. Furthermore, whole-genome collinearity within the *DsMYB* family was examined and visualized using the Advanced Circos tool.

### 2.5 Conserved motifs, domain architecture, and gene structure analysis

Conserved domains within DsMYB protein sequences were identified using the NCBI Batch CDD-search. Gene structural features, including exon-intron organization and untranslated regions (UTRs), were extracted. Finally, we performed a comprehensive analysis by integrating conserved motifs, conserved domains, gene structures, and the *DsMYB* phylogenetic tree using TBtools.

### 2.6 *Cis*-elements of promoters analysis

The *cis*-acting elements in the promoter sequences of *DsMYB* genes from *D. styracifolium* were predicted using PlantCARE (https://bioinformatics.psb.ugent.be/webtools/plantcare/html/), and visualization of the analysis results were perf-ormed through TBtools.

### 2.7 Expression patterns of *DsMYB* genes

Total RNA was extracted from the roots, stems, and leaves of *D. styracifolium* using a commercial RNA isolation kit. RNA quality, concentration, and integrity were verified through UV spectrophotometry and agarose gel electrophoresis, respectively. Qualified RNA was reverse-transcribed into first-strand cDNA using a reverse transcription kit for subsequent expression analysis. Eight highly expressed *DsMYB* genes were selected for quantitation. Primers were designed using Primer Premier (**Supplementary Table 1**), and qRT-PCR was performed under the following conditions: 95 °C for 30 s; followed by 40 cycles of 95 °C for 5 s and 60 °C for 30 s. To ensure reliability, three independent biological replicates were used for each tissue, with EF-1α serving as the internal reference gene. Additionally, the 2^−ΔΔCt^ method was employed to calculate the expression levels of *DsMYB* genes (Livak and Schmittgen, 2001).

## 3 Results

### 3.1 Gene identification and physicochemical property analysis

Using a combined gene mining strategy of hmmsearch and blastp, followed by manual curation, a total of 161 protein sequences containing MYB or MYB-like repeats were identified in the *D. styracifolium* genome. These sequences were designated *DsMYB001* to *DsMYB161*. Based on the number of domain repeats, these MYB members were categorized into four canonical groups: R2R3-MYB (134 genes), 1R-MYB (21 genes), 3R-MYB (5 genes), and 5R-MYB (1 gene).

The lengths of the DsMYB proteins ranged from 88 to 1654 amino acid, with molecular weights spanning 10.27 to 179.91 kDa. Analysis revealed that the isoelectric points (pI) of 89 members were acidic (pI < 7.0), while 72 were basic (pI > 7.0). Stability indices indicated that the majority of DsMYB proteins are unstable; notably, only seven members exhibited instability indices below 40 (31.98 - 38.81). All DsMYB proteins were determined to be hydrophilic, as indicated by grand average of hydropathicity (GRAVY) values below zero. Additionally, aliphatic amino acid indices (52.18 - 83.32) suggested high thermal stability. Furthermore, subcellular localization prediction indicated that 93% (150 members) were localized in the nucleus, while 6% (10 members) in chloroplasts, and one member (*DsMYB081*) in the cytoplasm (**Supplementary Table 2**).

### 3.2 Phylogenetic analysis

**Figure 1:**
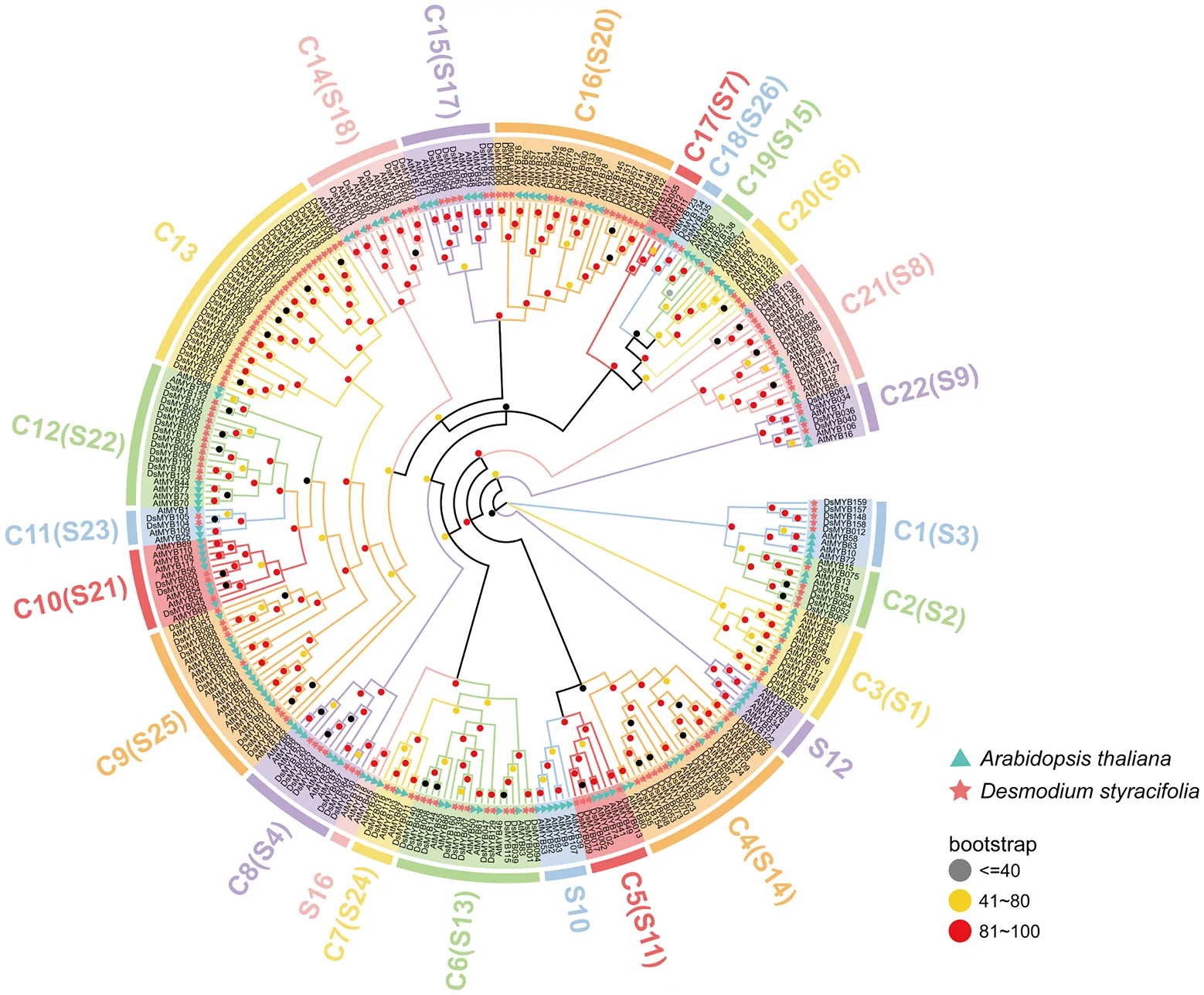
Phylogenetic tree constructed based on MYB proteins from *D. styracifolium* and *Arabidopsis thaliana*. (Leaf level: triangles represent *A. thaliana*, pentagrams represent *D. styracifolium*; branches: each colored module indicates one subgroup. ‘C’ denotes subgroups specific to *D. styracifolium*, and ‘S’ denotes subgroups from *A. thaliana*).

To further elucidate the evolutionary relationships within the *DsMYB* family, a phylogenetic analysis was conducted using the 161 identified DsMYB proteins and 131 previously characterized *AtMYB* members. The results showed that the DsMYB proteins cluster into 25 distinct subgroups (**Figure 1**), with *D. styracifolium* and *A. thaliana* appearing together in 23 subclades.

Notably, the largest subgroup C13 was unique to *D. styracifolium* comprising 30 members; this suggested that these genes might have experienced evolution independently within this species. The other subgroups with following size were identified as C16, C9, and C4, containing 24, 22, and 21 members, respectively. Conversely, subgroups S12, S10, and S16 consisted exclusively of *AtMYB* genes, indicating a potential loss of these orthologs during the evolution of *D. styracifolium*. Such species-specific gene gains and losses within the MYB family may have contributed to functional divergence.

According to previous studies, subgroup S6 members in *A. thaliana* (*AtMYB75*, *AtMYB90*, *AtMYB113*, and *AtMYB114*) regulate anthocyanin biosynthesis, while subgroup S7 members (*AtMYB11*, *AtMYB12*, and *AtMYB111*) modulate flavonol biosynthesis (Dubos et al., 2010; Schilbert and Glover, 2022). Consequently, subgroups C20 (S6) and C17 (S7) identified in this study could be associated with these metabolic pathways; however, further experimental verification is required to confirm these regulatory roles.

### 3.3 Chromosomal localization and collinearity analysis

**Figure 2:**
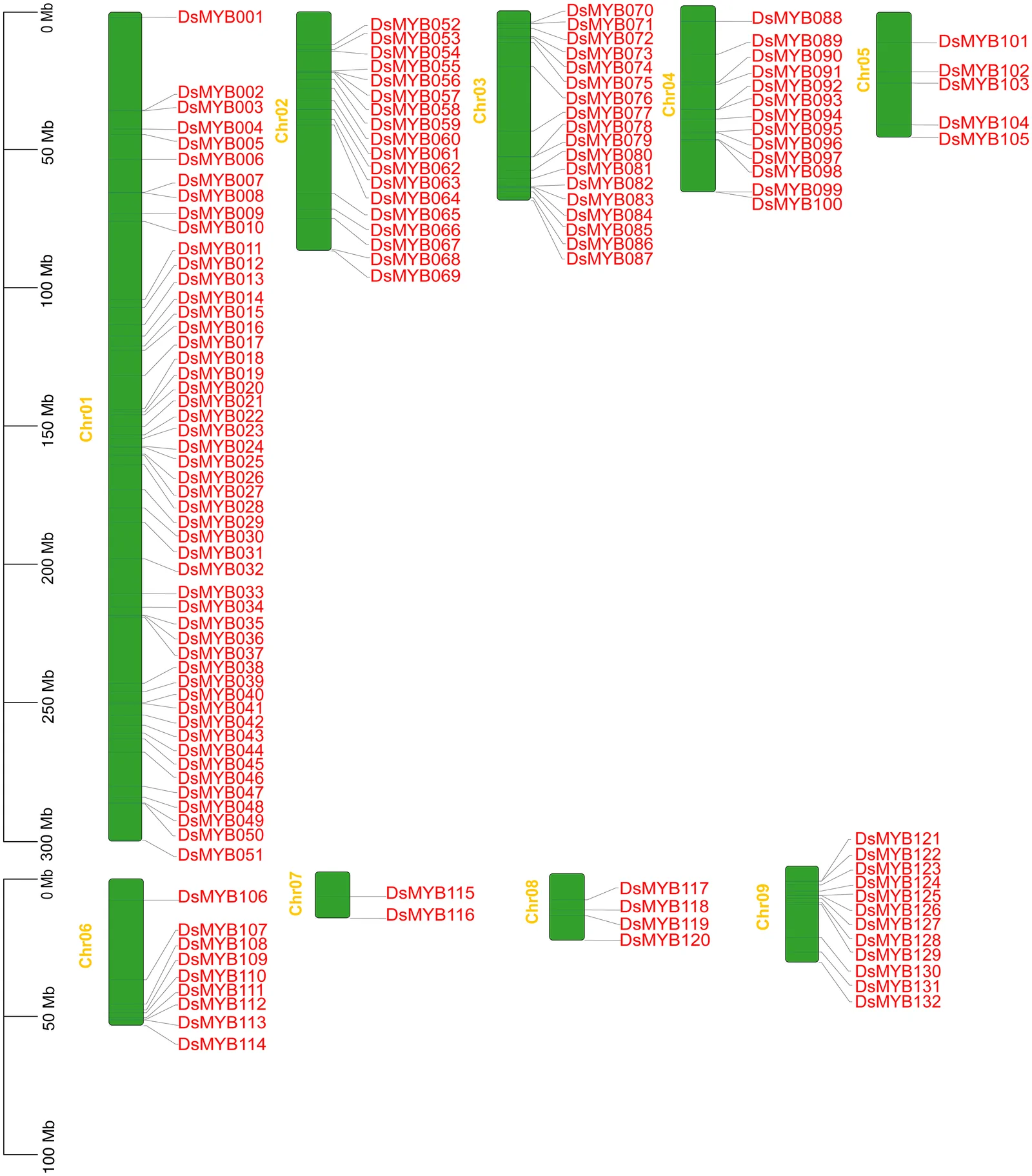
Distribution characteristics of MYB genes on chromosomes of *D. styracifolium*, ‘Chr’ denotes the chromosome.

Chromosomal localization analysis revealed that 132 of the 161 *DsMYB* genes are unevenly distributed across nine chromosomal pseudomolecules, while the remaining 29 *DsMYB* genes are located on unplaced scaffolds and cannot be assigned to any chromosome (**Figure 2**). Chromosome 1 contained the highest density of *DsMYB* genes, harboring 51 members, followed by chromosome 2 and 3, which contained 18 genes each. The remaining chromosomes comprise between 2 to 13 *DsMYB* genes, further illustrating an uneven distribution pattern across the genome.

**Figure 3:**
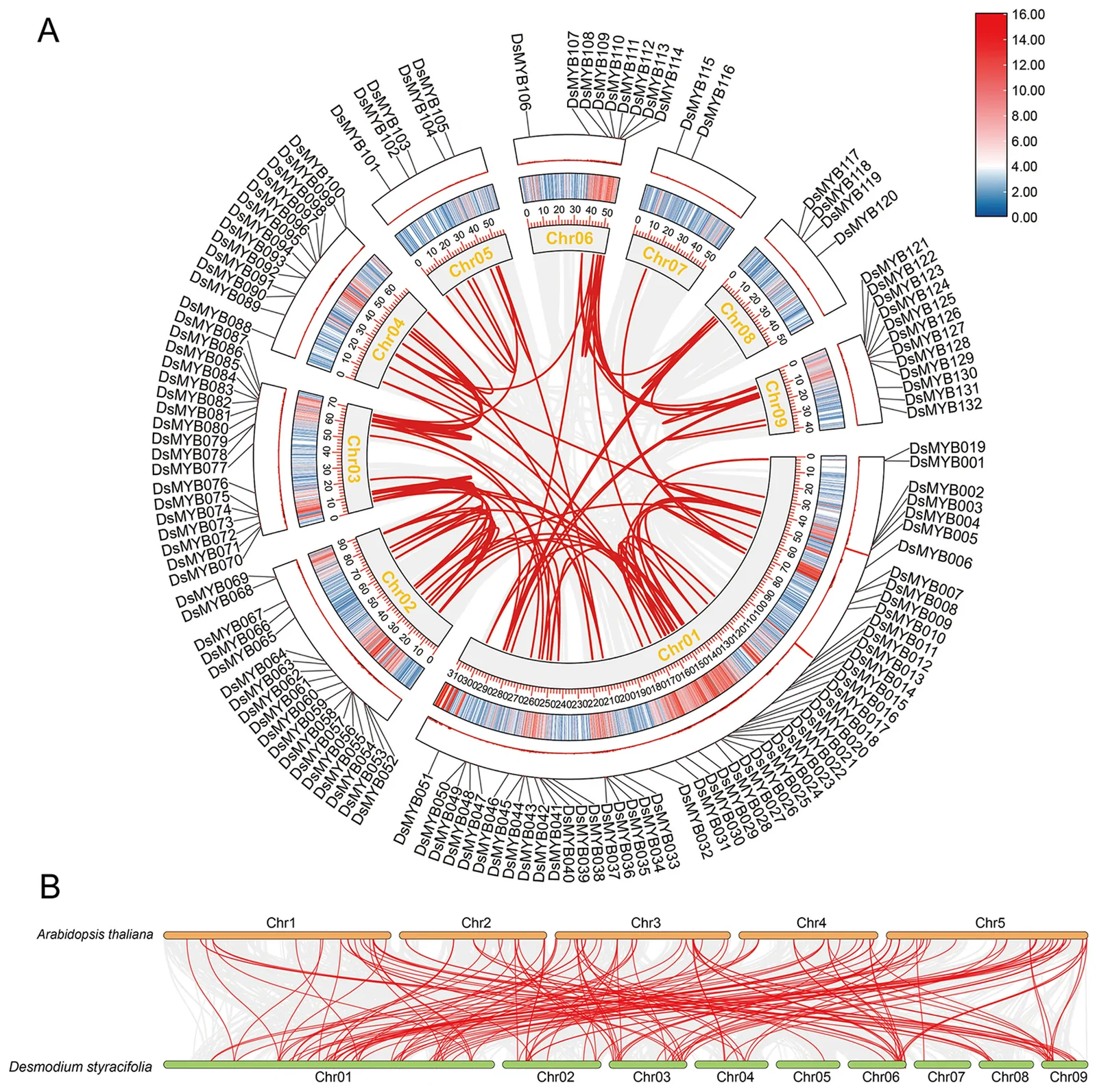
Chromosomal collinearity analysis of *DsMYB*s. **(A)** Collinearity analysis of *DsMYBs* within the *D. styracifolium* genome (Circos plot), The gray lines in the background represent collinear blocks within the genome of *D. styracifolium*, while the red lines indicate syntenic *DsMYB* gene pairs. **(B)** Interspecific collinearity analysis of *DsMYBs* between *D. styracifolium* and *A. thaliana.*

Collinearity analysis (**Figure 3A**) indicated that *DsMYB* genes exhibited high levels of collinearity among different chromosomes in *D. styracifolium*, with collinear gene pairs detected on all chromosomes. Within the *D. styracifolium* genome, 132 *DsMYB* genes formed 103 pairs through whole-genome duplication (WGD). This finding suggests that WGD served as a primary driver for the expansion and evolution of the *DsMYB* family in this species.

To further investigate the evolutionary characteristics of the *DsMYB* family, interspecific collinearity was analyzed between *D. styracifolium* and the model plant *A. thaliana*. The results (**Figure 3B**; **Supplementary Table 3**) revealed that 84 *DsMYB* members formed 141 collinear gene pairs with *AtMYB* orthologs. Conserved chromosomal linkage relationships are depicted by red lines in **Figure 3B**. These extensive interspecific connections represent retained collinear segments, indicating that the MYB families of both species may have originated from ancient duplication events. These results established a chromosomal framework for exploring the functional conservation and evolutionary lineage of these genes.

### 3.4 Analysis of conserved motifs, domain architecture, and gene structure

**Figure 4:**
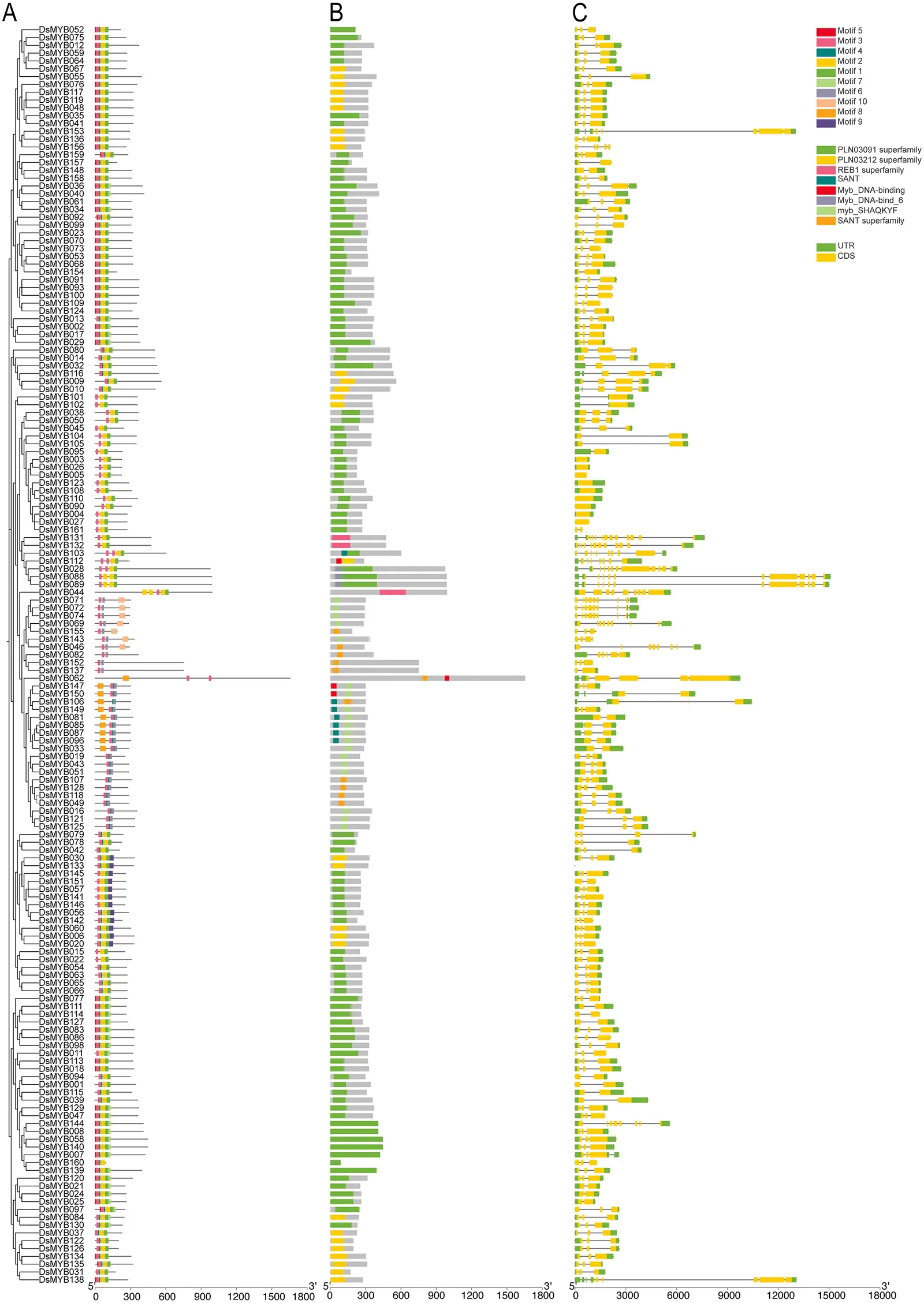
Evolutionary and structural analysis of *DsMYBs*. **(A)** Distribution of conserved motifs in *DsMYBs*, where each motif is represented by a distinct color. **(B)** Conserved domains identifed in *DsMYBs.* **(C)** Exon–intron structures of *DsMYBs*, UTR and CDS indicate non-translated regions and coding sequences, respectively.

To characterize the structural features of *DsMYB* transcription factors, conserved element analysis was performed against these 161 *DsMYB* members, which predicted a total of 10 motifs (**Figure 4A**; **Supplementary Table 4**). Most *DsMYB* members contained motif 2, 3, 4, and 5, which were sequentially arranged at the N-terminus; this configuration indicates that these motifs are relatively conserved within the family. Meanwhile, all DsMYB proteins comprise four essential domains: Myb_DNA, PLN03091, REB1, and SANT (**Figure 4B**).

Gene structure analysis revealed that the number of exons ranged from 1 to 13, while the number of introns varied from 0 to 12 (**Figure 4C**). Generally, genes within the same subfamily exhibited similar structural organizations. However, notable variations in exon counts were observed within specific subfamilies. For instance, while most R2R3-MYB members contain 3 exons, *DsMYB131* harbors 13 exons. These results suggested that exon gain and loss events may have occurred during the evolutionary divergence of *DsMYB* genes.

### 3.5 Analysis of *Cis*-regulatory elements within gene promoters

C*is*-acting elements within the promoter region are crucial for the regulation of gene expression. To investigate the regulatory mechanisms of *DsMYB* genes, the 2000 bp sequences upstream of the initiation codon were analyzed. A total of 3106 *cis*-acting elements were identified, among which the TATA-box and CAAT-box were the most widely distributed. Functional classification of these elements revealed that light-responsive elements were overwhelmingly dominant, with 2418 in total (**Supplementary Table 5**).

**Figure 5:**
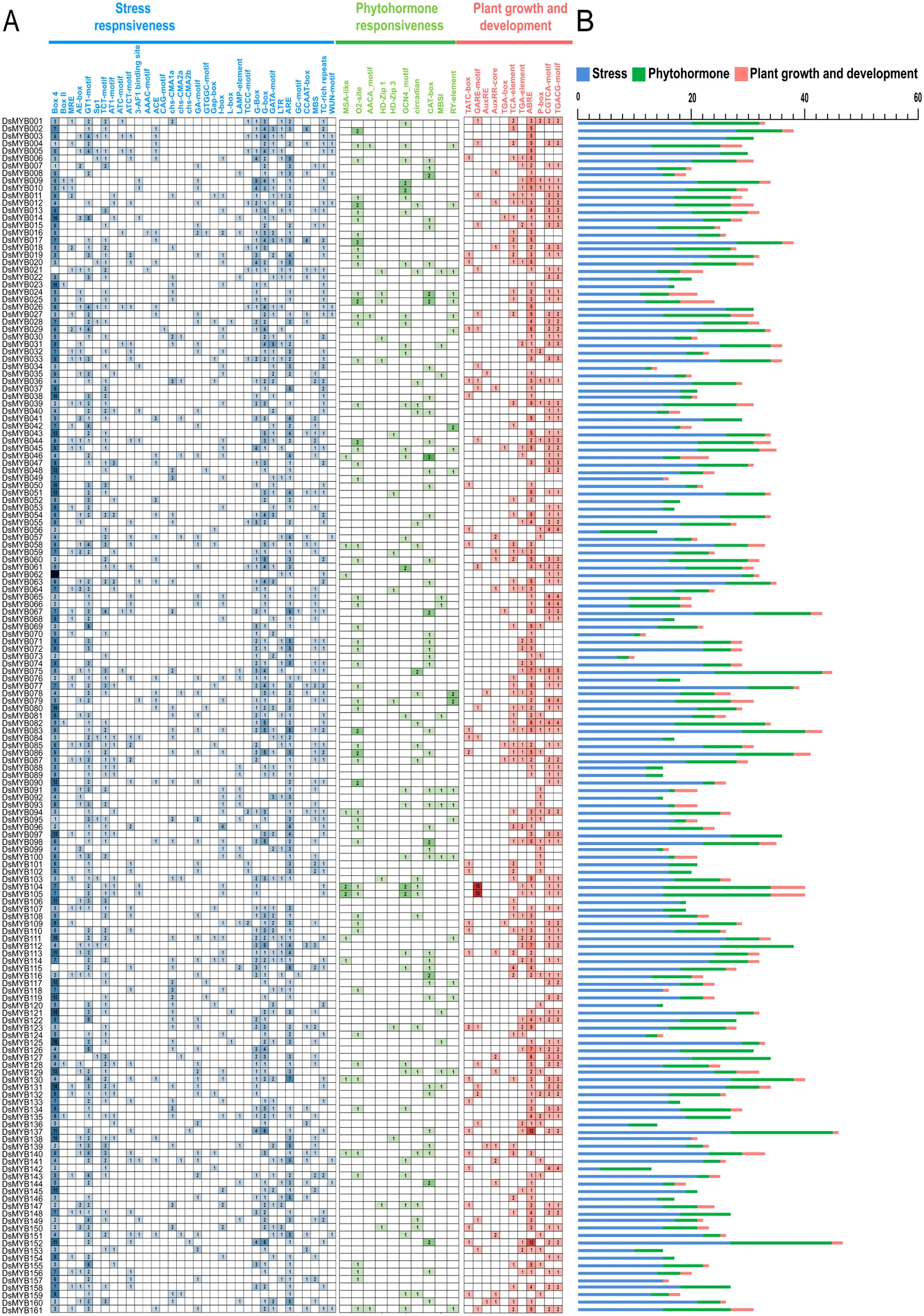
Analysis of *cis*-acting elements in the *DsMYB* gene family. **(A)** Distribution of *cis*-acting elements in 161 *DsMYB* genes. **(B)** Bar chart showing the number of *cis*-acting elements related to abiotic stress response, hormone response, and plant growth and development in *DsMYB* genes.

The high frequency of Box-4 (986) and G-box (409) elements strongly suggest that the *DsMYB* family might function as a hub within the regulatory network integrating environmental light signals, circadian rhythms, and stress response. This configuration indicates a potential role in modulating plant physiological status during diurnal or seasonal changes. Additionally, numerous abscisic acid-responsive elements (ABRE, 389) and methyl jasmonate-responsive elements (TGACG-motif, CGTCA-motif, 342) were detected, implying that *DsMYB* genes are involved in hormone-mediated defense and survival. The co-occurrence of G-box and ABRE elements further suggested that DsMYB proteins might participate in “light-stress cross-tolerance”, such as the simultaneous response to high light and drought stress (**Figure 5**).

Furthermore, several low-frequency elements were identified, including anaerobic induction elements (ARE, 298), low-temperature responsive elements (LTR, 45), seed-specific regulatory elements (GCN4_motif and RY-element), and circadian control elements. Notably, MYB binding sites (MBSI) involved in the regulation of flavonoid biosynthesis were also present (**Supplementary Figure S1**). Collectively, these results demonstrated that the promoter regions of *DsMYB* genes are enriched with diverse elements associated with environmental and developmental signals. These findings highlight the potential involvement of the *DsMYB* family in various biotic and abiotic stress responses and corroborate the central role of *DsMYB* transcriptional factors in integrating light signaling with stress defense.

### 3.6 Expression patterns of the *DsMYB* gene family

Gene expression patterns are often associated with its function. To characterize the expression profiles of *DsMYB* genes, transcriptome data from five tissues: roots, stems, leaves, flowers and fruits, were determined. The results revealed that 161 *DsMYB* genes were expressed in at least one tissue, with 136 members exhibit expression across all tissues. FPKM values ranged from 0 to 148, and the highest relative expression levels were observed in the stems.

**Figure 6:**
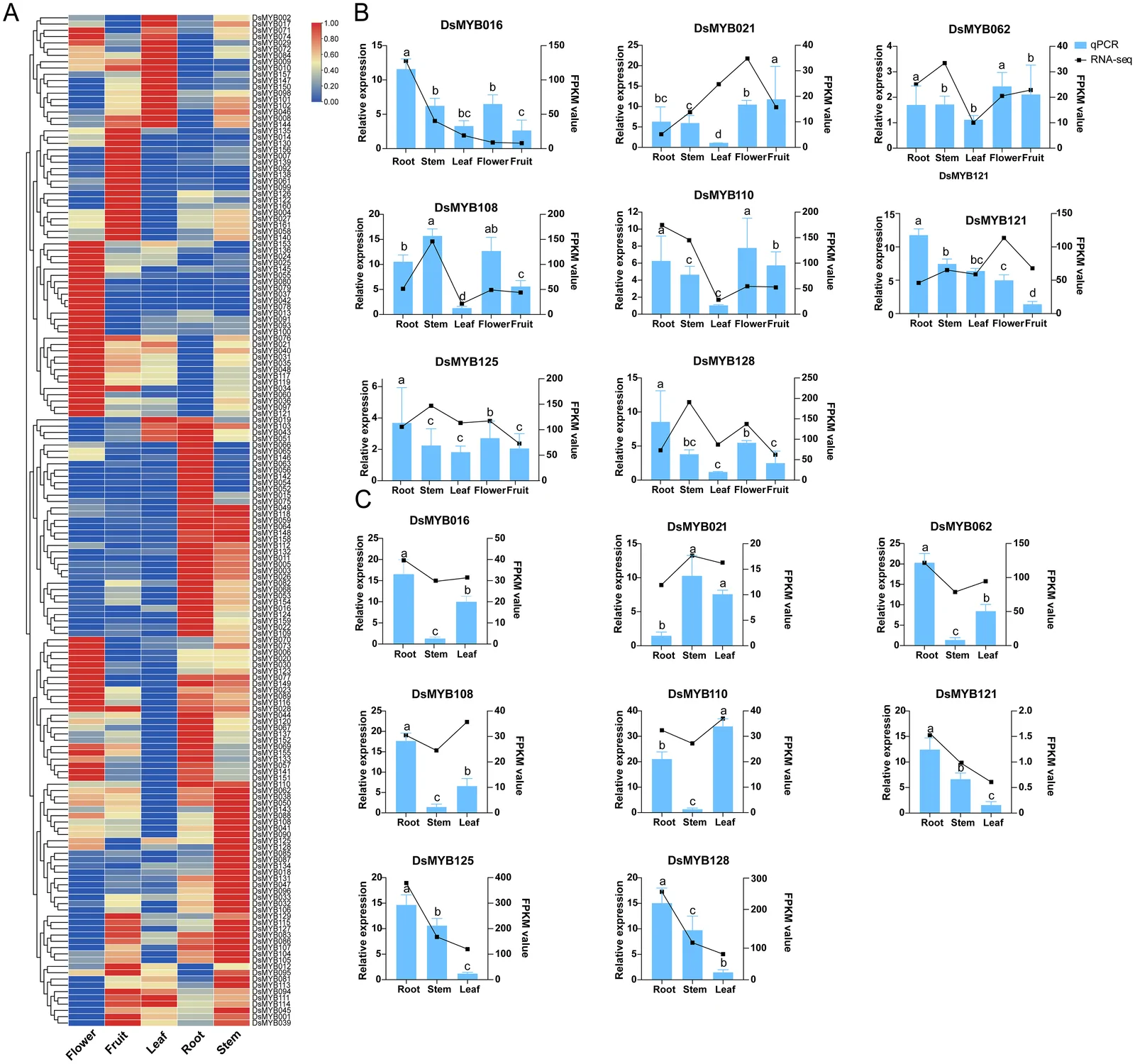
Expression pattern analysis of *DsMYB* gene family members. **(A)** Expression pattern analysis of *DsMYB* gene family members in *D. styracifolium*. FPKM values were derived from pooled samples of three biological replicates per tissue. **(B)** qRT-PCR analysis of relative expression of *DsMYB* in different *D. styracifolium* tissues. **(C)** qRT-PCR analysis of relative expression of *DsMYBs* under highlight treatment of *D. styracifolium* at 72 h. Error bars represent the mean ± SD of three independent experiments. Different lowercase letters indicate significant differences among different tissues as determined by one-way analysis of variance (ANOVA) followed by Duncan’s multiple range test (*p* < 0.05).

Further investigation identified several *DsMYB* genes with tissue-specific expression patterns. For example, *DsMYB125* and *DsMYB128* showed high relative expression across all five tissues. In contrast, *DsMYB037*, *DsMYB042*, *DsMYB078*, and *DsMYB079* were specifically expressed in flowers (**Figure 6A**). These divergent gene expression patterns suggested potential functional specialization during the growth and development of *D. styracifolium*.

To validate the transcriptome findings, eight differentially expressed *DsMYB* genes were selected for quantitative real-time PCR (RT-qPCR) analysis. Consistent with the transcriptomic data, *DsMYB016*, *DsMYB121*, *DsMYB125*, and *DsMYB128* were predominantly expressed in roots, while *DsMYB021* and *DsMYB062* showed high expression in flowers. Additionally, *DsMYB108* exhibited significantly higher expression in stems compared to other tissues (**Figure 6B**). Overall, the expression trends of seven genes were in agreement with the transcriptome data.

Furthermore, the response of the family to abiotic stress was evaluated by subjecting *D. styracifolium* plants to 72 hours of continuous high-light treatment. Due to withering of flowers and fruits during the treatment period, RT-qPCR analysis was restricted to vegetative organs. The experimental results indicated that high-light treatment significantly induced the expression of most *DsMYB* genes, with the most pronounced induction occurring in the leaves (**Figure 6C**; **Supplementary Figure S2**).

## 4 Discussion

### 4.1 Basic characteristics and evolutionary analysis of the MYB gene family in *D. styracifolium*

The MYB transcription factor family represents one of the largest gene families in plants, playing pivotal roles in regulating diverse biological processes including plant growth and development, secondary metabolism, defense, and environmental stress responses. In this study, a total of 161 *DsMYB* transcription factors were identified for the first time at the whole-genome level in *D. styracifolium*. The size of the MYB genes family varies considerably across plant species: 244 in *Glycine max* (Du et al., 2012), 205 in *Gossypium hirsutum* (cotton) (He et al., 2016), 116 in *Prunus sibirica L.* (Zhao et al., 2024), and 97 in *Morinda officinalis* (Li J. et al., 2023). The abundance of MYB genes in *D. styracifolium* falls within the typical range observed for these angiosperms. Such lineage-specific variation in gene number is primarily driven by differences in the frequency of whole-genome duplication (WGD) and segmental duplication events experienced during species evolution (Ding et al., 2024).

Chromosomal mapping revealed that 132 *DsMYB* genes are unevenly distributed across nine chromosomes, with chromosome 1 harboring the highest gene density (51 members). Similar non-random chromosomal distribution has been documented in other plant lineages, such as *Artemisia* (Peng et al., 2025) and *Cocos nucifera* L (Si et al., 2024). Extensive studies have confirmed that segmental duplication serves as the primary engine driving R2R3-MYB family expansion in dicots. In *Glycine max*, genome-wide segmental duplication coupled with tandem duplication jointly accounted for MYB gene expansion (Du et al., 2012), a mechanism further corroborated by whole-genome analyses in *Chenopodium quinoa*, *Spatholobus suberectus*, and cowpea (*Vigna unguiculata*) (Ding et al., 2024; Qin et al., 2023; Xia et al., 2026).

Phylogenetic analysis further resolved the MYB proteins of *D. styracifolium* into 25 distinct subgroups. Notably, the C13 subgroup formed a species-specific clade unique to this species, suggesting independent evolutionary trajectories and potential functional specialization within this species. Analogous species-specific clade differentiation has been observed in the MYB gene family of melon (Xu et al., 2024), supporting the paradigm that MYB family undergo lineage-specific divergence to adapt to distinct ecological niches during evolutionary processes.

### 4.2 Functional analysis of promoter *Cis*-acting elements

Analysis of promotor *cis*-acting element revealed that *DsMYB* regulatory regions are heavily enriched with motifs responding to diverse environmental and developmental cues. Light-responsive elements (predominantly Box-4 and G-box) predominated, closely accompanied by abundant abscisic acid (ABA) and methyl jasmonate (MeJA) responsive elements. This architecture mirrors the MYB element distribution patterns reported in paper mulberry (Zhu et al., 2025), implying that *DsMYB* may serve as a core regulatory hub coordinating the crosstalk between light signaling and stress-defense pathways. This regulatory logic appears conserved across legumes and other non-model species as: in pigeon pea (*Cajanus cajan*), most *CcR2R3-MYB* genes respond to multiple environmental stresses (Li H. et al., 2023), and in mung bean (*Vigna radiata*), MYB transcription factors are in indispensable for growth, development, and abiotic stress adaptation (Li et al., 2026). Similarly, genome-wide promoter profiling of the Dof family in pitaya (Hylocereus) revealed an enrichment in ABA, MeJA-, and light-responsive elements (Alam et al., 2024). Together, these findings reinforce the notion that diverse transcription factor families in non-model plants adopt a conserved promoter-based regulatory mode to integrate multiple environmental signals.

The frequent co-occurrence of G-box and ABRE elements strongly suggests that the *DsMYB* family functions as an important molecular link bridging ABA-dependent stress response pathways with light and circadian clock regulatory networks (Mahapatra et al., 2025; Zhao et al., 2022). Conversely, the scarcity of low-temperature-responsive (LTR) elements aligns with the ecological preference of *D. styracifolium* for warm climates and its inherent weak cold tolerance, implying that *DsMYB* play an auxiliary role rather than functioning as a core executors in low-temperature responses. In addition, the widespread distribution of flavonoid-related MBS elements, anaerobic induction AREs, and circadian clock-related elements underscores the multi-faceted capacity of *DsMYB* to simultaneously integrate developmental and environmental signals. Under normal light/dark cycles, light signals and circadian rhythms likely drive baseline *DsMYB* expression to maintain physiological homeostasis. Upon encountering environmental stresses (e.g., drought, cold, or flooding), corresponding hormonal signals (ABA, JA) and stress signals are dynamically activated. These signaling molecules modulate *DsMYB* expression patterns by binding to promoter motifs such as ABRE, TGACG motifs, ARE, and LTR elements, thereby coordinating resource allocation and prioritizing plant survival.

### 4.3 MYB-mediated regulation of C-glycosylated flavone biosynthesis

*D. styracifolium* is distinguished from most medicinal plants by its high enrichment in bioactive flavone C-glycosides, notably schaftoside and vicenin, yet the MYB-mediated transcriptional regulatory network underlying this metabolic hallmark has remained largely unexplored. A recent breakthrough in duckweed (*Lemna turionifera*) identified a P1-like R2R3-MYB transcription factor, LtP1L, as a master orchestrator of CGF biosynthesis: LtP1L directly activates the expression of flavanone 2-hydroxylase (F2H) and C-glycosyltransferase (CGT), the two gateway enzymes for CGF formation, and its overexpression increased CGF levels six-fold, whereas CRISPR-mediated knockdown reduced CGF content by 74.3% (Wang et al., 2024). In maize, the P1 gene analogously governs the accumulation of the C-glycosyl flavone maysin (Zhang et al., 2003). Phylogenetically, LtP1L was reported to cluster with *AtMYB11*, *AtMYB12*, and *AtMYB111* in subgroup S7 (Wang et al., 2024). In our phylogenetic tree, the Arabidopsis S7 clade members (*AtMYB11/12/111*) resolve within subgroup C17 of *D. styracifolium* MYBs, indicating that C17 is the orthologous clade to the flavonol-regulating S7 subgroup. Given this phylogenetic affinity and the fact that several C17 members harbor MBSI elements (a flavonoid biosynthesis-related *cis*-motif) in their promoters, we propose that C17 members are the most promising candidates for governing CGF biosynthesis in *D. styracifolium*, and their functional characterization represents a logical next step.

## 5 Conclusions

In this study, a total of 161 *MYB* transcription factors were identified at the whole-genome level in *D. styracifolium*. Their conserved domains, chromosomal distribution, phylogenetic relationships, gene duplication patterns, and expression profiling were systematically characterized. Furthermore, by integrating phylogenetic clustering, promotor *cis*-element features, and tissue-specific as well as high-light stress expression profiles, the potential regulatory functions of *DsMYB* genes in flavonoid biosynthesis and stress responses were predicted. Collectively, this study systematically elucidates the fundamental features of the *DsMYB* gene family, providing a solid theoretical foundation and crucial genetic targets for future in-depth functional characterization, molecular breeding, and synthetic biology applications in this leguminous medicinal plant.

## Data Availability

The datasets presented in this study can be found in online repositories. The names of the repository/repositories and accession number(s) can be found in the article/**Supplementary Material**.
