# Supplementary material for: Genome-wide identification and expression analysis of the MYB transcription factor family in Desmodium styracifolium
Source: Front Plant Sci. 2026 Sep 3;17:1938247. doi: 10.3389/fpls.2026.1938247 (PMC13582623; doi:10.3389/fpls.2026.1938247)
Supplement: Supplementary file 1 [file DataSheet1.docx]

**Additional file:**

**Figure S1**. The cis-elements of 161 *DsMYBs* promoters. Promoter sequences (-2,000 bp) of *DsMYBs* were analyzed using PlantCARE. Different shapes and colors represented different elements.

**Figure S2**. Hightlight treatment expression profiles and phylogenetic tree of all identified MYB members in *D. styracifolium*.
